# Medial stabilised total knee arthroplasty achieves comparable clinical outcomes when compared to other TKA designs: a systematic review and meta-analysis of the current literature

**DOI:** 10.1007/s00167-020-06358-x

**Published:** 2020-11-27

**Authors:** Sohail Nisar, Kashif Ahmad, Jeya Palan, Hemant Pandit, Bernard van Duren

**Affiliations:** 1 Leeds Institute of Rheumatic and Musculoskeletal Medicine, University of Leeds, Chapel Allerton Hospital, Leeds, UK; 2 Bradford Royal Infirmary, Bradford, UK; 3 Leeds Teaching Hospitals NHS Trust, Leeds, UK; 4 Academic Department of Trauma and Orthopaedics, Leeds General Infirmary, University of Leeds, Leeds, UK

**Keywords:** Total knee replacement, Total knee arthroplasty, Medial pivoting, Medial stabilised

## Abstract

**Purpose:**

The purpose of this study was to perform a systematic review and meta-analysis to compare clinical and patient-reported outcome measures of medially stabilised (MS) TKA when compared to other TKA designs.

**Methods:**

The Preferred Reporting Items for Systematic Review and Meta-Analyses algorithm was used. The Cochrane Central Register of Controlled Trials, MEDLINE, EMBASE, and EMCARE databases were searched to June 2020. Studies with a minimum of 12 months of follow-up comparing an MS TKA design to any other TKA design were included. The statistical analysis was completed using Review Manager (RevMan), Version 5.3.

**Results:**

The 22 studies meeting the inclusion criteria included 3011 patients and 4102 TKAs. Overall Oxford Knee Scores were significantly better (*p* = 0.0007) for MS TKA, but there was no difference in the Forgotten Joint Scores (FJS), Western Ontario and McMaster Universities Osteoarthritis Index (WOMAC), Knee Society Score (KSS)-Knee, KSS-Function, and range of motion between MS and non-MS TKA designs. Significant differences were noted for sub-group analyses; MS TKA showed significantly worse KSS-Knee (*p* = 0.02) and WOMAC (*p* = 0.03) scores when compared to Rotating Platform (RP) TKA while significantly better FJS (*p* = 0.002) and KSS-knee scores (*p* = 0.0001) when compared to cruciate-retaining (CR) TKA.

**Conclusion:**

This review and meta-analysis show that MS TKA designs result in both patient and clinical outcomes that are comparable to non-MS implants. These results suggest implant design alone may not provide further improvement in patient outcome following TKA, surgeons must consider other factors, such as alignment to achieve superior outcomes.

**Level of evidence:**

III.

**Electronic supplementary material:**

The online version of this article (10.1007/s00167-020-06358-x) contains supplementary material, which is available to authorized users.

## Introduction

Knee kinematics are driven by a complex interaction of the tibiofemoral and patellofemoral joints with the supporting passive and active soft-tissue structures. Following total knee arthroplasty (TKA), it has been shown that the kinematics of the knee are different from what is seen in the native undiseased knee [2, 20]. Abnormal kinematics contribute to restricted knee flexion, reduced quadriceps efficiency, inferior functional outcome, and increased pain after TKA [5].

Since the introduction of the modern bicondylar TKA concept, designs have focussed on the recreation of tibial–femoral roll-back and stability in the sagittal plane using dished bearing surfaces or cam-post mechanisms. With observations showing a “medial pivot”-type behaviour of the natural knee [14, 15, 23, 27, 36], the medial pivot/medial stabilised (MS) concept was developed. The MS design aims to better reproduce the tibial–femoral kinematics observed in the healthy knee more closely. Typically, MS TKA designs have an asymmetric liner and femoral component with a spherical or single radius medial femoral condyle [8]. The geometry of the components in the medial compartment has an increased congruency providing increased sagittal stability while laterally the less congruent articulation permits the lateral condyle to roll and slide posteriorly with flexion of the knee [6].

Since the first generation of medial stabilised designs, The Advance Medial Pivot (AMP) (MicroPort Orthopedics Inc, Arlington, TN) knee launched in 1998 and the Medial Rotating Knee (MRK) (MatOrtho, UK) in 2001, the use of the MS concept had gained increasing traction. Currently, to our knowledge, a further seven designs have been introduced based on the MS design concept: GMK Sphere (Medacta, Castel San Pietro, Switzerland), Evolution MP (MicroPort Orthopedics Inc.), Alumina MP (Kyocera, Kyoto, Japan), SAIPH (MatOrtho), FINE Knee (Teijin Nakashima medical), K-Mod dynamic congruence (Gruppo Bioimpianti, Peschiera Borromeo, Milan, Italy), and the Persona Medial Congruent (MC) (Zimmer Biomet, Warsaw, IN).

There are several short-term follow-up studies of MS designs presented in the literature as well as a number of mid- to long-term follow-up studies on the first-generation MS implants [7–9, 13, 29]. Many of these studies present excellent results of MS design implants; however, the majority are retrospective and include varying forms of bias [8]. A number of systematic reviews and meta-analyses of MS designs have been published which have shown revision rates similar to other designs [13, 46]. However, there is less evidence looking at clinical outcomes in the presence of MS implant designs and it remains unclear if patients experience a benefit in outcomes.

By recreating more physiological knee kinematics, it is thought MS TKA will improve clinical outcomes. However, although there are numerous reviews reporting on survival of MS TKA, there are very few reviews investigating if MS TKA improves clinical outcomes. There is only a single meta-analysis previously which included only two studies in their analysis comparing MS TKA to PS TKA [46]. This current paper presents a comprehensive, up to date, systematic review and meta-analysis of available literature. It compares clinical and patient-reported outcome measures (PROMs) of the MS TKA design when compared to other TKA designs in patients undergoing TKA to test the hypothesis that MS TKA implants achieve improved clinical outcomes.

## Methods

The study protocol was registered with PROSPERO 2020 CRD42020171600. Available from: https://www.crd.york.ac.uk/prospero/display_record.php?ID=CRD42020171600.

The protocol for this systematic review was created prior to data extraction and was guided by the Preferred Reporting Items for Systematic Reviews and Meta-Analyses (PRISMA) checklist and algorithm [35]. MEDLINE, EMBASE, and EMCARE databases were searched. The Cochrane Central Register of Controlled Trials (CENTRAL) for RCTs, including ongoing trials was also searched. The following search strategy was used:

“total knee replacement*” OR “total knee joint replacement*” OR “total knee prosthe*” OR “total knee arthroplast*” OR “Knee Arthroplast*” OR “knee joint replacement*” OR “knee replacement*” OR “TKR” OR “TKA” OR “TJA” AND “medial* stabili#ed” OR "medial pivot" OR “medial-pivot” OR “medial* conforming” OR “ball and socket” OR “ball-and-socket” OR “MRK” OR “ADVANCE medial pivot” OR “SAIPH” OR “GMK Sphere” OR “MicroPort Evolution” OR “K-Mod” AND “outcome*” OR “measure*” OR “assess*” OR “score*” OR “scoring” OR “surviv*”

References of included studies and related reviews were checked to determine if further studies were available.

Inclusion criteria were established following the PICO (Population Intervention Comparison Outcomes) approach. Population: Adults (over 18) undergoing knee arthroplasty. Intervention: TKA using a MS design implant. Comparator: TKA using a conventional design implant. Outcomes: The primary outcomes were all clinical function scores and PROMs: Forgotten Joint Score (FJS), Knee Society Score (KSS)-Knee, KSS-Function, Oxford Knee Score (OKS), Western Ontario and McMaster Universities Arthritis Index (WOMAC), knee range of motion (ROM).

Only papers available in English were included. MEDLINE, EMBASE, and EMCARE databases were searched using the Healthcare Databases Advanced Search (HDAS) search tool with the results merged with The CENTRAL search result. Any duplicates were removed. Titles and abstracts were screened for relevance prior to full inspection independently by two investigators (SN, BvD). Any discrepancies between the independent investigators were referred to a third investigator (HP) for arbitration.

Randomised control trials, case–control, and case-series with a comparative control were included in this analysis. Data were extracted using a standardised data collection protocol. As with study assessment for inclusion, an arbitrator was consulted regarding any discrepancies. In addition to the outcomes listed above, the following data were recorded: a) Demographics: Population studied, Age, Gender, Implant (manufacturer, type, design), side, indication b) Study characteristics: study design, data collection period, number of subjects, randomisation, blinding, allocation concealment, funding, country of origin.

### Assessment of methodological quality

Risk of bias was assessed using the Critical Appraisal Skills Programme (CASP) tools [48] for risk of bias to standardise assessment of the included trials as well as case–control and case-series. The studies were graded as low, medium, or high risk. The Grading of Recommendations Assessment, Development and Evaluation (GRADE) was used to assess the quality of the body of evidence for each of the selected outcomes [18]. Using GRADE, one of four levels of evidence or “certainty in evidence or quality” is assigned: high = further research is very unlikely to change confidence in the estimate; moderate = further research is likely to have an important effect on confidence in the estimate and may change the estimate; low = further research is very likely to have an important effect on confidence in the estimate and is likely to change the estimate. Very low quality: The estimate is very uncertain. Evidence from randomised controlled trials rate high quality and, because of residual confounding, evidence that includes observational data starts at low quality.

### Statistical analysis

The extracted data were analysed using the statistical software Review Manager version 5.3 (Cochrane, London, United Kingdom). Means and standard deviations (SD) were extracted from each study for meta-analysis. Patients with MS TKA were compared to patients with other implant designs based on functional outcomes (FJS, KSS-Knee, KSS-Function, OKS, WOMAC, ROM). As there are numerous other TKA designs with varying knee kinematics, MS TKA was compared individually to PS TKA, CR TKA and RP TKA as well as a presentation of MS TKA compared to all other TKA designs.

Where SDs were not provided in the published manuscript these were then calculated either from supplemental data [37] or from the provided confidence intervals, standard errors, and p-values using the methods described in the Cochrane Handbook (Chapter 7.7.3.3 [22]).

Heterogeneity between studies from clinical or methodological diversity was considered likely and as such a random-effects model was used. In all studies, *p* < 0.05 was considered statistically significant. The consistency of results across the pooled studies was estimated using the calculated *I*^2^ statistic to measure heterogeneity, representing the percentage of variation in our meta-analysis caused by heterogeneity rather than by chance. A value of less than 30% was interpreted as a low heterogeneity and above 75% as high heterogeneity [22].

## Results

**Fig. 1:**
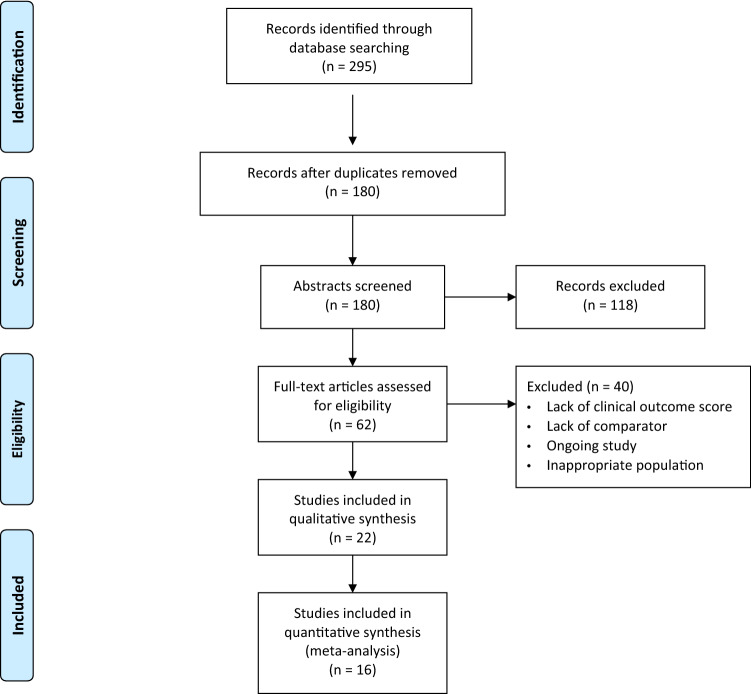
Prisma flow diagram giving an overview of the literature search & review

The literature search yielded 295 results of which 115 duplicates were removed. 180 remaining abstracts were screened and 118 were excluded as they did not meet the inclusion criteria. The remaining 62 full-text records were reviewed. 22 studies from ten countries meeting the inclusion criteria [1, 2, 4, 10–12, 16, 17, 24–26, 30–32, 34, 37, 39–42, 45, 47] were identified (see PRISMA flowchart in Fig. 1). Of these, eight were RCTs [4, 11, 12, 16, 24, 26, 30, 39], three prospective cohort studies [31, 32, 40], and 11 retrospective studies [1, 2, 10, 17, 25, 34, 37, 41, 42, 45, 47]. In total, the studies included 4,102 knees in 3,011 participants with 3,911 knees remaining after accounting for participant dropout, loss to follow-up, and subgroup selection (see supplementary file). The overall mean age was 70 years (age range 26–89 years) with the mean age for the cohorts being: MS 69.5, all comparators 69.5, Posterior Stabilised (PS) 71.1, Cruciate Retaining (CR) 68.3, Mobile Bearing/Rotating Platform (RP) 66.4 (units in years). The overall mean follow-up was 52.6 months (SD 32.7). Six studies were excluded from subsequent meta-analysis owing to insufficient information (two studies reported medians rather than means [26, 45], three studies reported delta scores only [11, 31, 47], and the remaining study did not report SD and lacked any further statistical detail to calculate these [40]) resulting in 16 studies being included in the meta-analysis.

### Cohorts & implants

The 22 studies reviewed included 22 cohorts of MS implant designs compared to between one and four cohorts using other implant designs resulting in a total of 51 cohorts of patients. A further single cohort was excluded as this cohort utilised a unicompartmental arthroplasty [45]. Three studies did not include full details of the manufacturer and implant type. Lee et al. [32] only specified design concept without either manufacturer or implant details for both MS and comparator cohorts. Both Gill et al. [17] and Pritchett [40] only specified manufacturer details for the comparator cohorts. Excluding these, 8 medial stabilised implants and 14 comparator implants were identified (see supplementary file). Taking into account the studies excluded for insufficient data, the meta-analysis included 33 cohorts (16 MP, 17 comparator design concepts [13 PS, two CR, two RP]) with seven medial stabilised implant designs and 19 comparator implants specified (Lee et al. [32] no implant data specified).

### Complications

Four studies did not report complications or lack thereof [4, 17, 40, 42], seven studies reported having no complications [10, 12, 26, 37, 39, 45, 47] and the remaining studies reported 62 complications (see supplementary file).

### Risk of bias

An overview of the Risk of bias assessments is shown in Table 1. 14 studies were graded low, 4 low/moderate, 3 moderate and 1 as moderate/high risk.

**Table 1:** Tables to demonstrate the study bias assessments using the CASP checklists [48]

CASP- Retrospective	Q1	Q2	Q3	Q4	Q5	Q6	Q7	Q8	Q9	Q10	Q11	BIAS
CR vs MP												
Nakamura [37]	Yes	Yes	Yes	Yes	Yes	A) Yes	Hi-Tech Knee 11 (CR) > Flat surface (FINE) especially in surgical time/ Blood loss/ ROM (flexion angle), knee pain	KSS-Knee: 7.20 [3.54, 10.86]. KSS-Function: − 2.60 [-10.68, 5.48]. ROM: 6.80 [0.40, 13.20]	Yes	No	Yes	Low
						B) Yes						
MB vs MP												
Choi [10]	Yes	No	Yes	Yes	Yes	A) Yes	RP > MB for demanding exercise. P value only. No odds ratio	KSS-Knee: − 2.20 [− 5.15, 0.75]. KSS-Function: 1.00 [− 2.47, 4.47]. WOMAC: 2.80 [-0.17, 5.77]. ROM: − 3.10 [− 7.70, 1.50]	Yes	No	Yes	Low
						B) Yes						
PS vs MP												
Anderson et al. [1]	Yes	No	Yes	Yes	Unclear	A) Yes	Significantly less patellofemoral complications with substitution of the PCL without a cam-and-post mechanism	ROM: − 3.00 [− 10.37, 4.37]	Yes	No	Yes	Moderate
						B) Yes						
Shakespeare et al. [42]	Yes	No	Unclear	Yes	Unclear	A) Yes	Comparable results for knee flexion at one year follow-up	ROM: − 2.00 [− 4.45, 0.45]	Yes	No	Yes	Moderate/ High
						B) Unclear						
Bae [2]	Yes	No	Yes	Yes	Yes	A) Yes	Comparable results for MP + PS in pain relief, function, radiographic results + complication rate	KSS-Knee: 1.00 [− 0.44, 2.44]. KSS-Function: − 1.40 [-3.15, 0.35]. WOMAC: − 1.50 [-2.79, − 0.21]. ROM: − 3.40 [− 6.90, 0.10]	Yes	Unclear	Yes	Low
						B) Yes						
Wautier and Thienpont [45]	Yes	Yes	Yes	Yes	Yes	A) Yes	No stability at 60degrees in any TKA. No differences in clinical outcome (Patient reported outcome)	A pilot study was carried to assess variance. This served as an. Estimate for the effect size and an appropriately power was calculated	Yes	No	Yes	Low
						B) Yes						
Samy [41]	Yes	No	Yes	Yes	Yes	A) Yes	MP > PS on FJS score (Pt reported outcome)- particularly on knee flexion and stability	FJS: 14.95 [4.01, 25.89]. ROM: 5.76 [0.17, 11.35]	Yes	No	Yes	Low
						B) Yes						
Indelli et al. [25]	Yes	No	Yes	Yes	Yes	A) Yes	Comparable short-term outcomes where reducing the level of intra-articular constraint did not have an overall negative effect. There is minimal increase in active ROM when a more anatomical medial congruent insert is used	OKS: 0.60 [0.24, 0.96]. ROM: 3.00 [0.19, 5.81]	Yes	No	Yes	Low
						B) Yes						
Gill et al. [17]	Yes	Unclear	Yes	Yes	Yes	Yes	MP > PS for knee flexion and satisfaction	FJS: 12.48 [3.61, 21.35]. KSS-Knee: 0.40 [− 0.63, 1.43]. ROM: 6.00 [4.67, 7.33]	No	Yes	Yes	Moderate
Yuan et al. [47]	Yes	Yes	Yes	Yes	Yes	A) Yes	No difference in post-operative midterm functional outcome or complication	WOMAC: − 0.48 [− 7.00, 6.04]	Yes	No	Yes	Low
						B) Yes						
Lin et al. [34]	Yes	No	Yes	Yes	Yes	A) Yes	MP achieved satisfactory short-term clinical outcomes, but not superior to PS prostheses. Persistent pain was an important risk factor of dissatisfaction in TKA	ROM-PS1: 0.30 [− 2.89, 3.49]. ROM-PS2: 0.70 [− 3.45, 4.85]	Yes	No	Yes	Low
						B) Yes						

CASP-Retrospective	Q1	Q2	Q3	Q4	Q5	Q6	Q7	Q8	Q9	Q10	Q11	Q12	BIAS
PS vs MP													
Lee et al. [34]	Yes	Yes	Yes	Yes	A) Yes	A) Yes	Comparable clinical and satisfaction	FJS: − 7.00[-16.03, 2.03]. KSS-Knee: 1.00 [− 5.10, 7.10]. KSS-Function: -2.00 [− 10.79, 6.79]. WOMAC: 3.00 [− 2.33, 8.33]	Unclear	No	No	Yes	Low/ Moderate
					B) Yes	B) Unclear							
Pritchett [40]	Yes	Yes	Yes	Yes	A) Yes	A) Yes	Bilateral knee arthroplasties preferred retention of their ACL and PCL, or substituted with the MP Prosthesis	Significant powered study in all intergroup comparisons to detect a large size effect	Yes	No	Yes	Yes	Low/ Moderate
					B) Yes	B) Unclear							
Kulshrestha et al. [31]	Yes	Yes	Yes	Yes	A) Yes	A) Yes	MP > PS for daily living activities. PS demonstrated better knee flexion. Equal satisfaction with both designs	FJS: 2.9 [− 4.10, 9.90]. KSS-Knee: 23 [15.56, 30.44]. KSS-Function: 0.8 [− 5.07, 6.67]. ROM: 15.4 [7.38, 23.42]	Yes	No	Unclear	Unclear	Low
					B) Yes	B) Yes							

CASP- RCT	Q1	Q2	Q3	Q4	Q5	Q6	Q7	Q8	Q9	Q10	Q11	Bias
CR vs MP												
French [6]	Yes	Yes	Yes	Unclear	Yes	Yes	MS > CR in QoL and FJS. Otherwise comparable	FJS: 16.10 [1.31, 30.89]. OKS: 1.20 [− 1.13, 3.53]. WOMAC: -2.80 [-6.88, 1.28]. ROM: 0.80 [-3.01, 4.61]	No	Yes	Yes	Low/ Moderate
MB vs MP												
Kim [30]	Yes	Yes	Yes	Yes	Yes	Yes	Long-term fixation/ survival rate comparable	KSS-Knee: -5.00 [-9.38, − 0.62]. KSS-Function: 0.00 [− 15.61, 15.61]. WOMAC: 7.00 [0.15, 13.85]. ROM: − 11.00 [− 21.24, − 0.76]	No	Yes	Unclear	Low
PS vs MP												
Hossain [24]	Yes	Yes	Yes	Yes	Yes	Yes	MP > PS in ROM (equal at 12 months) + clinical function	OKS: − 2.90 [− 6.46, 0.66]. KSS-Knee: 7.70 [− 0.24, 15.64]. KSS-Function: 3.40 [− 5.71, 12.51]. WOMAC: − 5.80 [− 14.08, 2.48]. ROM: 14.80 [8.47, 21.13]	Yes	Yes	Yes	Low
Ishida et al [26]	Yes	Yes	Yes	Unclear	Yes	Yes	MP equal to PS at 4–5 year follow-up. Results suggested that differences in insert design only could not improve clinical benefits at midterm follow-up	Adequately powered study based of previous study sample size and power analysis	No	Yes	Yes	Low/ Moderate
Benjamin et al. [4]	Yes	Yes	Unclear	Unclear	Yes	Yes	Equally good results in clinical outcome. No statistically significant difference in gait analysis	OKS: − 0.80 [− 8.97, 7.37]. KSS-Knee: − 2.10 [− 18.97, 14.77]	Yes	Yes	Yes	Moderate
Nishitani et al. [39]	Yes	Yes	Yes	Yes	Yes	Yes	Comparable results for patient-reported outcomes and ROM at 2 years follow-up	KSS-Knee: − 6.00 [13.57, 1.57]. KSS-Function: 1.20 [− 8.26, 10.66]. ROM: 1.80 [− 5.83, 9.43]	No	Yes	Yes	Low
EdelsteIn et al. [12]	Yes	Yes	Yes	Yes	Yes	Yes	MS > PS sagittal plane stability in mid-flexion. Increased satisfaction (non-validated questionnaire)	FJS: 2.50 [− 19.48, 24.48]. OKS: 2.13 [− 3.58, 7.84]. KSS-Knee: 2.10 [− 4.09, 8.29]. KSS-Function: 4.00 [− 7.27, 15.27]. ROM: − 3.50 [− 9.35, 2.35]	Unclear	Yes	Yes	Low
Dowsey et al. [11]	Yes	Yes	Yes	Yes	Yes	Yes	Comparable clinical results. MS design associated with better patient-reported outcomes	OKS: − 1.4 (− 10.4, 7.6). KSS-Knee: 0.6 (− 9.5, 10.7). KSS-Function: − 3.4 (− 15.9, 9.1). WOMAC: − 6.3 (− 16.5, -3.9)	No	Yes	Yes	Low

### Meta-analyses

#### FJS

**Fig. 2:**
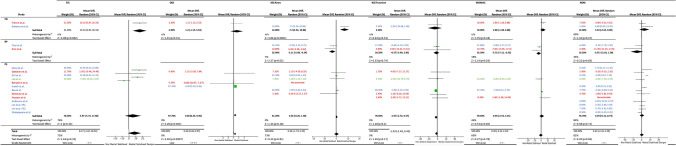
Forest plot and  GRADE Assessment for FJS, OKS, KSS-Knee, KSS-Function, WOMAC and ROM values of medial stabilised vs. non-medial stabilised cohorts. *SD* standard deviation, *CI* confidence interval, *CR* cruciate retaining, *PS* posterior stabilising, *RP* rotating platform. NB: PS1 & PS2 represent 2 cohorts of PS designs in a single study [34]. Red = RCT, Green = Prospective Cohort Study, Blue = Retrospective Cohort Study

Meta-analysis of the five eligible studies did not show an overall significant difference in FJS (*p* = 0.10) (Fig. 2). Three of the included studies reported a significant difference in favour of the MS implants over the comparator [16, 17, 41]. Subgroup analysis showed a significant improvement of FJS in MS TKA over CR TKA, however, this was based on a single study [16].

#### OKS

Overall, there was a significant difference in favour of MS TKA (*p* = 0.0007) (Fig. 2). Sub-group analysis showed no significant differences in both CR and PS analyses. Hossain et al. [24] reported the OKS on the 60-point scale, rather than the 48-point scale, and so could not be included in the analysis.

### KSS-Knee

Overall, there was no significant difference between the MS and non-MS groups, however, sub-group analysis showed a significant difference in both CR and RP analyses (*p* < 0.05); however, these only included 1 and 2 studies per group, respectively (Fig. 2).

### KSS-Function

Overall, there was no significant difference between the MS and non-MS groups as was the case for the sub-analysis groups (Fig. 2). Four further studies included in the systematic review but not the meta-analysis [11, 26, 31, 40], reported no significant difference in KSS-Function scores.

#### WOMAC

The six studies included in the meta-analysis showed no significant difference overall. Sub-group analysis showed an improved post-operative WOMAC score for the RP TKA group over the MS TKA group which was significant (*p* = 0.03) (Fig. 2).

#### ROM

Meta-analysis included 15 studies and showed no significant differences between overall and sub-group analyses (Fig. 2). Three of the included studies reported a significant difference in post-operative ROM between MS and non-MS cohorts (two in favour of MS implants [17, 31], and one in favour of an RP implant [30].

## Discussion

The key takeaway point from this study is that there is no clear consensus in favour of either MS or non-MS groups; however, sub-group analysis suggests that MSTKA performs better than PS and CR designs but worse than RP designs. Meta-analysis showed a significant advantage of MSTKA in OKS (*p* = 0.0007) whereas all other measures (FJS, KSS-Knee, KSS-Function, and ROM) showed no significant difference. Of the implant designs compared to MSTKA, the majority (twelve) were PSTKAs with two comparing CRTKA and RPTKA, respectively. An overview of the analysis of the sub-groups based on comparator designs across the outcome measures is given in Table 2. Significant differences were noted for sub-group analyses with MSTKA having superior KSS-Knee (*p* = 0.0001) and FJS (*p* = 0.002) scores over CRTKA. Significantly inferior KSS-Knee (*p* = 0.02) and WOMAC scores (*p* = 0.03) were noted for MS TKA when compared to RP TKA, and superior OKS when compared to PSTKA (*p* = 0.001).

**Table 2:** Table showing an overview of sub-analyses for TKA designs compared to MP TKA (e.g. CR designs showed an inferior overall FJS on meta-analysis than MP TKA)

	FJS	OKS	KSS-Knee	KSS-Function	WOMAC	ROM
CR	Inferior^a, b^	Inferior^b^	Inferior^a, b^	Superior^b^	Inferior^b^	Inferior^b^
RP	-	-	Superior^a^	Superior	Superior^a^	Superior
PS	Inferior	Inferior^a^	Inferior	Superior	Inferior	Inferior

^a^A significant difference on meta-analysis

^b^A score with only one published outcome included in the meta-analysis

Only one previous meta-analysis has been undertaken to assess clinical and PROMs following MS TKA in comparison to non-MS designs [46]. Young et al. [46] only included two papers, both of which are included in the present analysis [2, 24]. Both these papers compared MS to PS TKA. The authors of the review found a significant difference in post-operative WOMAC values favouring the MS group and superior KSS values in favour the non-MS group. In contrast, the present study found no significant differences in KSS or WOMAC scores when comparing MS TKA to non-MS TKA nor PS TKA. Young et al. [46] included the old KSS from Hossain et al. [24] with the new KSS from Bae et al. [2] in the same analysis, however, these scores cannot be numerically correlated as such the KSS values from Hossain et al. have been excluded in the present analysis.

Although significant differences in outcome scores were noted between implant design cohorts in this study, it is important to consider if these observed changes represent a clinically noticeable difference. The mean difference in OKS in this study was 0.64 points (Fig. 2). The minimal clinically important difference (MCID) of the OKS has been investigated by Beard et al. [3] who suggest a 5-point difference as the MCID and a 4-point difference as the minimal detectable change (MDC). Similarly, the mean difference in KSS-Knee scores was 3.86 & 2.37 for MS-TKA in comparison to CR-TKA and RP-TKA subgroups, respectively (Fig. 2). Lee et al. [33] in their study, concluded the MCID for the KSS-Knee to be 5.3–5.9. Therefore, the statistically significant differences in scores noted in this study for these PROMS may not necessarily be clinically relevant.

Conversely, ceiling effects associated with the use of PROMs may limit their ability to detect significant differences. Clinical outcomes following orthopaedic surgery are often assessed using PROMs, however, as techniques and surgical procedures improve, ceiling effects become more apparent. Ceiling effects which occur when a high proportion of patients achieve either the best or worst score making it difficult to distinguish between patients. If 15% or more patients attain the highest score a ceiling effect of the scoring system becomes a concern [19]. Jenny et al. found the OKS to have a ceiling effect of 33% [28]. Conversely, Harris et al. using a large UK population, did not demonstrate a ceiling effect with the OKS [20]. Van Hemert et al. found the KSS score was unable to differentiate between high functioning UKR patients and patients with a TKR [21]. Of interest is that in the present study, the OKS reached statistical significance but the FJS did not; the FJS has demonstrated a much lower ceiling effect of 16% [43] which is considerably lower than for the OKS.

MS TKA having no clear advantage/disadvantage in clinical or patient outcome measures, when comparing to all other implant designs, may be a result of a medial pivot motion in TKA not correlating with improved clinical outcome. Studies correlating intra-operative medial pivot patterns with post-operative outcomes have been conflicting. Nishio et al. [38], using the PFC Sigma (Depuy, Warsaw, IN, USA) implant, demonstrated patients with a medial pivot pattern identified using intraoperative CT-based navigation achieved better post-operative outcomes. However, Warth et al. [44], in a similar study, used intra-operative digital sensor technology to correlate intra-operative kinematic patterns with post-operative outcomes. The authors used the Triathlon® (Stryker, Inc., Mahwah, NJ) implant and observed no difference in post-operative outcomes between those patients with a medial pivot pattern and those without [44].

There were limitations associated with this systematic review and meta-analysis. The lack of Level 1 RCTs addressing this topic was, as was the case with previous reviews [13, 46], a primary limitation; and therefore, the inclusion of cohort and case–control studies within our review. Accepting that this increases the risk of bias, we have undertaken a thorough CASP assessment and using the GRADE criteria assigned one of four levels of evidence or “certainty in evidence or quality” (see Table 1). The definition of medial pivot design may be a limitation in that there are numerous designs that can/or cannot be classified as having geometry where the medial compartment has increased congruency providing increased sagittal stability while laterally, the less congruent articulation permits the lateral condyle to roll and slide posteriorly with flexion of the knee resulting in a ‘medial pivot’ motion with flexion. For the purposes of this analysis, designs with fully congruent medial condylar contact and a less congruent lateral articulations have been included. However, despite the design requiring a fully congruent medial contact for inclusion, no restriction was placed on the lateral condyle, meaning we may not necessarily be reviewing directly comparable pivot motion. There was a large variation in reported outcomes between studies which is reflected by the heterogeneity measures which have been reported for both overall and subgroup analyses. Variable statistical data were reported with some studies not including SDs and as such these were calculated from p-values and confidence intervals. However, some studies were still excluded due to insufficient statistical data. This compounded the problems associated with low numbers. Similarly, sub-group analyses comparing MP to CR as well as MP to RP prostheses were limited owing to only two studies using the CR prosthesis and two using the RP prosthesis being available for analysis. Only English language studies were included as such relevant literature in non-English languages may have been missed.

## Conclusion

There is no clear advantage or disadvantage in clinical- or patient reported outcome measures when comparing MS implants to all other implant designs. This systematic review and meta-analysis has shown that MS TKA designs result in both patient and clinical outcomes that are comparable to non-MS implant designs. Some significant differences were noted to suggest MS TKA resulted in superior outcomes when compared to PS TKA. Comparisons between MS TKA to CR and RP TKA were limited by the number of included studies but suggest MS TKA may be superior to CR but inferior to RP in terms of clinical outcomes and highlight the need for further investigation. Ultimately the heterogeneity noted for the outcome measures in this analysis suggests that there is no clear correlation between biomechanical constraints included in implant designs and clinical outcomes. These results suggest implant design alone may not provide further improvement in patient outcome following TKA, surgeons must consider other options, such as alignment to achieve superior outcomes.

## Electronic Supplementary Material

Below is the link to the electronic supplementary material.

Supplementary material 1 (DOCX 122 kb)
